# Real-world burden of comorbidities in US patients with psoriatic arthritis

**DOI:** 10.1136/rmdopen-2017-000588

**Published:** 2017-12-28

**Authors:** Kamal Shah, Maria Paris, Lillian Mellars, Arun Changolkar, Philip J Mease

**Affiliations:** 1 GPS Innovation and GPS US, EMD Serono, Billerica, Massachusetts, USA; 2 Drug Safety, Celgene Corporation, Summit, New Jersey, USA; 3 Swedish Medical Center and University of Washington School of Medicine, Seattle, Washington, USA

**Keywords:** psoriatic arthritis, dmards (biologic), dmards (synthetic), cardiovascular disease

## Abstract

**Objectives:**

We assessed comorbidities associated with psoriatic arthritis in a broad cohort of US-insured adult patients using the Truven Health Analytics MarketScan Database.

**Methods:**

Prevalence and incidence rates were assessed for 28 comorbid conditions among adult patients in the MarketScan database with a diagnosis of psoriatic arthritis and having two or more health claims for psoriatic arthritis between 1 July 2008 and 31 July 2015. Findings were compared with those of a similar, previously published analysis of patients with psoriasis.

**Results:**

Among a total of 186 552 patients with a diagnosis of psoriatic arthritis, 94 302 had two or more health claims for psoriatic arthritis during the study period and were included in the comorbidity analysis. The prevalence and incidence rates of the most common comorbidities were 47.5% and 35.0% for hyperlipidaemia, respectively; 47.3% and 31.3% for hypertension; 21.2% and 15.4% for depression; 20.2% and 13.5% for type 2 diabetes mellitus; and 16.6% and 12.4% for fibromyalgia. Patients with psoriatic arthritis had notably higher incidence rates of uveitis, fibromyalgia, osteoporosis, Crohn’s disease and non-alcoholic liver disease than patients with psoriasis.

**Conclusion:**

This observational retrospective analysis using the MarketScan database provides real-world health claims data on the prevalence and incidence of comorbidities in a large US patient population with psoriatic arthritis. The observed high cardiometabolic comorbidity rates align with those reported in the literature and may help healthcare providers in the comprehensive management of patients with psoriatic arthritis.

**Key messages**

**What is already known about this subject?**

- Psoriatic arthritis is associated with comorbidities that can influence treatment decisions and management strategies.
- Prevalence and incidence rates of comorbidities associated with psoriatic arthritis have not been fully characterised.

**What does this study add?**

- This observational retrospective analysis using the MarketScan database provides real-world health claims data on the prevalence and incidence of comorbidities in a large US patient population with psoriatic arthritis.

**How might this impact on clinical practice?**

- Understanding the prevalence and incidence of comorbidities can aid healthcare professionals in the comprehensive management of patients with psoriatic arthritis.

## Introduction

Psoriatic arthritis is a chronic, inflammatory musculoskeletal disease that has multiple manifestations, including psoriasis. Increasing evidence supports the association between psoriatic arthritis and multiple comorbidities, including obesity, metabolic syndrome, cardiovascular disease, cerebrovascular disease and peripheral vascular disease.^1^ Some studies have provided the rate of selected comorbidities in patients with psoriasis^2–4^; however, fewer studies have been conducted in patients with psoriatic arthritis. In particular, rates of comorbidities in US patients with psoriatic arthritis have not been fully characterised.

Comorbidity rates may be determined using various approaches, including randomised trials or studies using postmarketing surveillance, registry or medical claims data; each approach has its strengths and weaknesses. The study populations included in randomised clinical trials often do not represent the broader, real-world populations.^5^ Postmarketing surveillance data consist of spontaneously reported adverse events that are often affected by under-reporting bias.^6–8^ Patient registries address some of these issues, providing more robust information, but they often capture data from studies with limited sample sizes and study durations.^9^

Medical insurance claims databases can be used to study large populations of patients afflicted with a specific disease and provide an effective means to assess comorbidity-related event rates in real-world populations. This study used a large insurance claims database to gain an understanding of the rate of comorbidities in a broad population-based cohort of adult patients with psoriatic arthritis.

## Methods

### Data source

Using the Truven Health Analytics MarketScan Database (Truven Health Analytics, an IBM company, Ann Arbor, Michigan, USA), health insurance claims from insured individuals were examined from 1 July 2008 through 31 July 2015. The database contains US administrative claims for commercially insured, working-age adults and their dependents, as well as individuals with Medicare supplemental insurance paid for by employers. Employer-provided data allow for tracking across health plans and, overall, contain administrative claims and eligibility records for approximately 230 million patient-lives since 1995.^10^ Enrolment records contain demographic information, including age, sex and geographical region. Medical claims files include inpatient, outpatient, facility and service claims records. The database is compliant with the Health Insurance Portability and Accountability Act and contains synthetic identifiers to protect the privacy of all patients and data contributors.

### Study population

Adult patients (≥18 years of age) who were diagnosed with psoriatic arthritis using the International Classification of Diseases, 9th Revision, Clinical Modification (ICD-9-CM) code 696.0x,^11^ with the first diagnosis claim between 1 July 2008 and 30 June 2014 and at least one additional psoriatic arthritis-related claim on or before 31 July 2015, were included in the study. The first diagnosis claim was set as the index date. Patients meeting the inclusion criteria are referred to as the psoriatic arthritis population. In addition, patients with psoriatic arthritis who met a minimum continuous health plan enrolment from 6 months before the index date through 6 months after the index date were selected and are referred to as the continuously enrolled population. Patients were studied until loss of insurance eligibility or the end of the study period.

### Study outcomes

The 28 outcomes chosen for this study reflect a broad spectrum of comorbid conditions (table 1). The ICD-9-CM codes chosen for the study outcomes were based on medical judgement or had been used in the literature previously. The ICD-9-CM codes for acute myocardial infarction (MI),^12^ stroke^13^ and depression^14^ have been validated, and the ICD-9-CM codes for infections have been used previously.^15^ Acute MI was determined using the primary diagnosis code for inpatient diagnoses. Stroke and infections were determined based on any inpatient diagnosis. Other outcomes were determined based on at least one diagnosis in any claim (see online supplementary table 1 for a complete list of codes and diagnosis claims considered).^12–17^

10.1136/rmdopen-2017-000588.supp1 Supplementary file 1

**Table 1:** Comorbid conditions selected for outcomes in patients with psoriatic arthritis

Comorbid conditions	
Acute myocardial infarction	Ischaemic stroke
Atherosclerosis	Non-melanoma skin cancer
Cardiac dysrhythmias	Non-alcoholic liver disease
Cerebrovascular diseases	Major depression
Chronic renal insufficiency	Major depression, suicide and suicidal ideation
Crohn’s disease	Osteoporosis
Congestive heart failure	Obesity
Depression	Peripheral vascular disease
Fibromyalgia	Solid tumours (including melanoma)
Gout	Stroke
Haematological malignancies	Type 2 diabetes mellitus
Hyperlipidaemia	Ulcerative colitis
Hypertension	Uveitis
Infections	
Ischaemic heart disease	

### Analysis

Continuous variables were summarised using mean and SD, and the discrete data were summarised using counts and percentages. Estimates for prevalence (%) and incidence (%) rates of the comorbidities were determined. Prevalence was defined as the proportion of occurrences of the specific comorbidity-related event during the study period divided by the total population. Incidence was defined as the proportion of new occurrences of the specific comorbidity divided by the population at risk for developing the comorbidity after the index date (ie, patients who had a claim for the comorbidity before the index date were excluded). In addition, the incidence (new occurrences of the specific comorbidity) per patient time (presented as per 100 patient-years) is provided, where patient time is the total years at risk that all patients contributed to the study. Included here are selected findings from an identically designed analysis of the MarketScan database performed in patients with psoriasis (identified through ICD-9-CM code 696.1)^18 19^ with the same index date and inclusion criteria that were used to identify patients with psoriatic arthritis in the current analysis. Portions of the findings in patients with psoriasis have been published.^19^ The ratio of the incidence rates per 100 patient-years for psoriasis^19^ and psoriatic arthritis was determined, along with the 95% confidence limits established using the Poisson distribution.

## Results

### Patients

The MarketScan database had 186 552 patients with at least one claim for a diagnosis of psoriatic arthritis between 1 January 2008 and 31 December 2014. Of these, 94 302 patients qualified for inclusion in the psoriatic arthritis population, and 47 438 patients met the continuous enrolment criteria. In the psoriatic arthritis population, 53.6% of the patients were female and the median age was 52 years. In each cohort, the numbers of patients diagnosed each year were similar, except for 2008 and 2014, which had fewer patients because the index date for the cohort was selected to allow the potential of 6 months of data before and after the index date (see online supplementary table 2 for demographic characteristics). The total follow-up for the psoriatic arthritis population was 150 626 patient-years, which represents an average of approximately 1.5 years per patient. Approximately one-third of patients were taking biologics at baseline, and 40% and 46% of patients were taking disease-modifying antirheumatic drugs and non-steroidal anti-inflammatory drugs, respectively (table 2).

**Table 2:** Therapeutic usage* among patients with psoriatic arthritis by drug class

Drug classification	Psoriatic arthritis population (n=94 302)	
	n†	%
Biologics‡	31 308	33.20
DMARDs§	38 099	40.40
NSAIDs	43 194	45.80
Steroids	56 915	60.35

*Treatments used on or after the index date (first diagnosis of psoriatic arthritis).

†Number of patients who received one or more therapies. Patients who received more than one therapy are included for each therapy class received.

‡Also known as biologic DMARDs.

§Include conventional oral DMARDs, including conventional synthetic DMARDs and the targeted synthetic DMARDs tofacitinib and apremilast.

DMARDs, disease-modifying antirheumatic drugs; NSAIDs, non-steroidal anti-inflammatory drugs.

### Comorbidities

The prevalence and incidence rates of the selected comorbidities in each cohort of patients with psoriatic arthritis are shown in table 3. Among patients in the psoriatic arthritis population, the most prevalent comorbidities (>10% of patients) were hyperlipidaemia (47.5%), hypertension (47.3%), depression (21.2%), type 2 diabetes mellitus (20.2%), fibromyalgia (16.6%), obesity (16.5%), ischaemic heart disease (11.6%) and cardiac dysrhythmias (10.4%). Incidence rates and incidence rates per 100 patient-years exhibited patterns of frequency ranking that were closely similar to prevalence rates. Table 4 presents the prevalence rates of comorbidities related to metabolic syndrome (eg, hyperlipidaemia, hypertension, type 2 diabetes mellitus, obesity) as observed currently and side by side with those in previously published reports,^20–22^ as well as in the general US adult population.^23–26^ Prevalence rates for these conditions were generally similar to prior reports in patients with psoriatic arthritis, except for obesity, which had a somewhat lower prevalence among the current patient cohort. Comorbidities related to metabolic syndrome were generally greater in the current cohort and in prior reports than in the general US population, with the exception of obesity, which is estimated to affect 36.5% of US adults.^25^

**Table 3:** Prevalence and incidence rates of comorbidities observed among patients with psoriatic arthritis

Comorbid condition†	Psoriatic arthritis population (n=94 302)	Continuously enrolled population* (n=47 438)	Psoriatic arthritis population (n=94 302)		Continuously enrolled population* (n=47 438)	
	Prevalence‡, n (%)		Incidence§, n (%)	Rate per 100 patient-years	Incidence§, n (%)	Rate per 100 patient-years
Hyperlipidaemia¶	44 768 (47.47)	23 811 (50.19)	23 156 (35.0)	27.11	8202 (30.02)	21.31
Hypertension¶	44 583 (47.28)	23 447 (49.43)	20 599 (31.25)	23.91	6773 (24.25)	17.15
Depression	19 995 (21.2)	10 656 (22.46)	12 853 (15.44)	10.16	5671 (14.34)	9.50
Type 2 diabetes mellitus¶	19 037 (20.19)	10 036 (21.16)	11 794 (13.49)	8.72	5558 (13.14)	8.57
Fibromyalgia	15 635 (16.58)	8306 (17.51)	10 453 (12.36)	8.07	4848 (11.96)	7.94
Obesity¶	15 510 (16.45)	8331 (17.56)	8742 (10.65)	7.05	3425 (8.69)	5.78
Ischaemic heart disease¶	10 918 (11.58)	6005 (12.66)	7256 (8.10)	5.19	3704 (8.44)	5.54
Cardiac dysrhythmias¶	9756 (10.35)	5510 (11.62)	6819 (7.73)	5.01	3022 (7.05)	4.66
Osteoporosis	8802 (9.33)	4665 (9.83)	6415 (7.16)	4.63	3017 (6.85)	4.56
Peripheral vascular disease	7298 (7.74)	4202 (8.86)	6275 (6.91)	4.40	3352 (7.49)	4.94
Infections**	7159 (7.59)	4058 (8.55)	5656 (6.26)	3.98	2967 (6.66)	4.37
Cerebrovascular disease¶	6903 (7.32)	3953 (8.33)	5478 (6.02)	3.84	2829 (6.31)	4.14
Major depression	6686 (7.09)	3655 (7.7)	4730 (5.14)	3.27	2391 (5.23)	3.43
Solid tumours, including melanoma	5963 (6.32)	3340 (7.04)	4343 (4.80)	3.04	2098 (4.69)	3.08
Gout	5673 (6.02)	3278 (6.91)	3757 (4.12)	2.62	1789 (3.97)	2.60
Non-alcoholic liver disease	5440 (5.77)	2930 (6.18)	3587 (3.88)	2.46	1780 (3.86)	2.53
Chronic renal insufficiency	4867 (5.16)	2637 (5.56)	3466 (3.85)	2.44	1777 (4.00)	2.63
Non-melanoma skin cancers	3707 (3.93)	2069 (4.36)	3041 (3.29)	2.08	1548 (3.36)	2.21
Congestive heart failure	3698 (3.92)	2157 (4.55)	2852 (3.07)	1.94	1539 (3.32)	2.18
Atherosclerosis¶	3355 (3.56)	1942 (4.09)	2803 (3.02)	1.91	1524 (3.3)	2.16
Uveitis	1466 (1.55)	790 (1.67)	949 (1.02)	0.64	424 (0.91)	0.59
Ulcerative colitis	1206 (1.28)	656 (1.38)	801 (0.86)	0.54	365 (0.778)	0.51
Haematological malignancies	1079 (1.14)	604 (1.27)	771 (0.82)	0.52	412 (0.88)	0.57
Crohn’s disease	1070 (1.13)	604 (1.27)	626 (0.67)	0.42	303 (0.64)	0.42
Acute myocardial infarction**††	524 (0.56)	292 (0.62)	506 (0.54)	0.34	281 (0.59)	0.39
Suicide and suicidal ideation	453 (0.48)	253 (0.53)	434 (0.46)	0.29	245 (0.52)	0.34
Any stroke**‡‡	445 (0.47)	255 (0.54)	420 (0.45)	0.28	234 (0.49)	0.32
Ischaemic stroke**‡‡	342 (0.36)	198 (0.42)	332 (0.35)	0.22	189 (0.40)	0.26

*Continuous enrolment with a minimum of 12 months: 6 months before the index date and 6 months after the index date.

†Includes all claims and any diagnosis field unless specified otherwise.

‡Prevalence is the proportion of occurrences for the specific comorbidity during the study period divided by the total population.

§Incidence is the proportion of new occurrences of the specific comorbidity divided by the population at risk for developing the comorbidity after the index date (ie, patients who had a claim for the comorbidity before the index date are excluded).

¶Medical conditions that can be associated with an increased risk of major adverse cardiac event.

**Includes only serious medical conditions (ie, those with an inpatient diagnosis).

††Acute myocardial infarction was based on the inpatient primary discharge diagnosis claims (ie, the primary discharge diagnosis).

‡‡Cases that likely represent a major adverse cardiac event.

**Table 4:** Prevalence of metabolic syndrome comorbidity in patients with psoriatic arthritis: comparison with literature and the general US population

Outcome, %	Continuously enrolled population	Merola *et al* 31*		Han *et al* 20†	Feldman *et al* 21‡	Husted *et al* 22§	US general population^23–26^
		Psoriatic arthritis and moderate to severe psoriasis	Psoriatic arthritis/minimal skin				
Hyperlipidaemia¶	47.5	55.3	53.8	27.8	34.6	20.7	12.1
Hypertension	47.3	51.0	49.7	28.5	35.8	37.1	25.0
Type 2 diabetes mellitus¶	20.2	21.2	20.0	11.3		12.0	9.4
Obesity	16.5	21.5	15.9	NA		30 (BMI ≥30 kg/m^2^)	36.5

*Merola *et al* reported on two groups of patients with psoriatic arthritis selected from the MarketScan database: patients with moderate to severe psoriasis and those with only minimal skin involvement.

†Han *et al* reported on 3066 patients with psoriatic arthritis who were selected from the PharMetrics’ Patient-Centric Database, which contains fully adjudicated medical service claims from health plans across the USA. Age-adjusted and sex-adjusted.

‡Feldman *et al* reported on 1230 US patients with psoriasis and comorbid psoriatic arthritis selected from the OptumHealth Reporting and Insights claims database.

§Husted *et al* reported on 611 patients with psoriatic arthritis selected from the University of Toronto Psoriatic Arthritis Clinic.

¶Merola *et al* reported on dyslipidaemia and diabetes versus type 2 diabetes mellitus specifically.

BMI, body mass index; NA, not applicable.

### Comparison with patients with psoriasis

Table 5 shows the comparison of the incidence rates of comorbidities per 100 patient-years for patients with psoriatic arthritis and an identically designed analysis of MarketScan data for patients with psoriasis.^19^ Incidence rates of fibromyalgia, gout and ulcerative colitis per 100 patient-years in patients with psoriasis were not included in the MarketScan psoriasis analysis publication. However, to provide comparison with all the comorbidities identified in the psoriatic arthritis analysis, we also examined and report here the incidence rates of these additional comorbidities in the psoriasis population (table 5). Compared with patients with psoriasis, those with psoriatic arthritis exhibited a higher incidence rate of nearly all comorbidities examined. Based on the analysis of the psoriatic arthritis population, the greatest disparities in incidence rate per 100 patient-years among patients with psoriatic arthritis versus patients with psoriasis were seen for uveitis (103% increase), fibromyalgia (77% increase), osteoporosis (75% increase), Crohn’s disease (58% increase) and non-alcoholic liver disease (50% increase) (table 5).

**Table 5:** Comparison of incidence rates of comorbidities observed among patients with psoriatic arthritis and psoriasis

Comorbidity	Full population (incidence per 100 patient-years)			Continuously enrolled population (incidence per 100 patient-years)		
	Psoriatic arthritis (n=94 302)	Psoriasis^19^ (n=469 097)	Ratio* (95% CI)	Psoriatic arthritis (n=47 438)	Psoriasis^19^ (n=292 999)	Ratio* (95% CI)
Hyperlipidaemia	27.11	21.70	1.25 (1.23 to 1.27)	21.31	18.81	1.13 (1.11 to 1.16)
Hypertension	23.91	16.39	1.46 (1.44 to 1.48)	17.15	13.49	1.27 (1.24 to 1.30)
Depression	10.16	7.78	1.31 (1.28 to 1.33)	9.50	7.36	1.29 (1.25 to 1.33)
Obesity	8.72	7.02	1.24 (1.22 to 1.27)	8.57	6.81	1.26 (1.22 to 1.30)
Fibromyalgia†	8.07	4.55	1.77 (1.73 to 1.81)	7.94	4.58	1.73 (1.68 to 1.79)
Type 2 diabetes mellitus	7.05	5.18	1.36 (1.33 to 1.39)	5.78	4.59	1.26 (1.21 to 1.30)
Cardiac dysrhythmias	5.19	4.47	1.16 (1.13 to 1.19)	5.54	4.65	1.19 (1.15 to 1.23)
Ischaemic heart disease	5.01	4.06	1.23 (1.20 to 1.27)	4.66	3.90	1.20 (1.15 to 1.24)
Osteoporosis	4.63	2.65	1.75 (1.70 to 1.80)	4.56	2.74	1.67 (1.60 to 1.73)
Infections	4.40	3.55	1.24 (1.21 to 1.27)	4.94	3.80	1.30 (1.25 to 1.35)
Peripheral vascular disease	3.98	3.42	1.16 (1.13 to 1.20)	4.37	3.62	1.21 (1.16 to 1.25)
Cerebrovascular disease	3.84	3.45	1.11 (1.08 to 1.14)	4.14	3.64	1.14 (1.09 to 1.18)
Non-alcoholic liver disease	3.27	2.18	1.50 (1.45 to 1.55)	3.43	2.13	1.61 (1.54 to 1.68)
Major depression	3.04	2.26	1.35 (1.30 to 1.39)	3.08	2.21	1.39 (1.33 to 1.46)
Solid tumours, including melanoma	2.62	2.53	1.03 (1.00 to 1.07)	2.60	2.49	1.05 (1.00 to 1.10)
Chronic renal insufficiency	2.46	1.87	1.31 (1.27 to 1.36)	2.53	1.95	1.29 (1.23 to 1.36)
Gout†	2.44	1.71	1.43 (1.37 to 1.48)	2.63	1.69	1.55 (1.47 to 1.64)
Non-melanoma skin cancers	2.08	2.35	0.88 (0.85 to 0.92)	2.21	2.51	0.88 (0.83 to 0.93)
Congestive heart failure	1.94	1.66	1.17 (1.12 to 1.22)	2.18	1.78	1.22 (1.16 to 1.29)
Atherosclerosis	1.91	1.87	1.02 (0.98 to 1.06)	2.16	2.02	1.07 (1.01 to 1.13)
Uveitis	0.64	0.32	2.03 (1.88 to 2.19)	0.59	0.32	1.86 (1.67 to 2.07)
Ulcerative colitis†	0.54	0.43	1.26 (1.17 to 1.36)	0.51	0.41	1.24 (1.11 to 1.39)
Haematological malignancies	0.52	0.45	1.14 (1.05 to 1.23)	0.57	0.46	1.25 (1.12 to 1.39)
Crohn’s disease	0.42	0.26	1.58 (1.45 to 1.73)	0.42	0.24	1.75 (1.55 to 1.99)
Acute myocardial infarction	0.34	0.49	0.68 (0.62 to 0.75)	0.39	0.56	0.70 (0.62 to 0.79)
Suicide and suicidal ideation	0.28	0.22	1.25 (1.12 to 1.39)	0.32	0.23	1.43 (1.24 to 1.65)
Any stroke	0.29	0.31	0.93 (0.84 to 1.03)	0.34	0.36	0.95 (0.83 to 1.09)
Ischaemic stroke	0.22	0.24	0.93 (0.83 to 1.05)	0.26	0.27	0.97 (0.83 to 1.12)

*Ratio is psoriatic arthritis/psoriasis incidence rates, where a ratio >1 indicates that patients with psoriatic arthritis have greater incidence.

†Gout, fibromyalgia and ulcerative colitis rates are not reported in the psoriasis MarketScan publication.^19^

## Discussion

The psoriatic disease process, with signs and symptoms that may involve joints (psoriatic arthritis) and skin (psoriasis), is driven by chronic, immune-mediated, systemic inflammation.^3 27^ Because underlying inflammatory processes may increase the risk of comorbid conditions,^3^ a better understanding of that risk may aid physicians in managing patients with psoriatic arthritis and psoriasis. In this study of a very large, population-based cohort of patients with psoriatic arthritis, the most common comorbidities were hyperlipidaemia, hypertension, depression, type 2 diabetes mellitus, obesity, ischaemic heart disease, fibromyalgia and cardiac dysrhythmias. These results in a US-insured population are comparable with previously published literature in patients with psoriatic arthritis and select comorbidities examined in other US and Canadian populations using other methodology.^20–22^

In patients with psoriatic arthritis, many of the most common comorbidities identified in the current analysis are related to metabolic syndrome, including hyperlipidaemia, hypertension, type 2 diabetes mellitus and obesity. The heightened risk of metabolic syndrome has been identified in patients with psoriatic disease,^4 28^ and metabolic syndrome, in turn, is a known risk factor for heart disease and stroke.^29^ The rates of these comorbidities have also been studied by others (table 4).^20–22^ Generally, prevalence magnitudes are similar among the reported analyses, although the results using the largest populations and the MarketScan database tend to have the highest prevalence rates. Compared with the general US adult population, prevalence of these comorbidities is markedly greater in patients with psoriatic arthritis (table 4). For example, diabetes mellitus is estimated to affect 9.4% of the general US population, and 90%–95% of these cases comprise type 2 diabetes mellitus.^23^ In the current study, 20.2% of patients with psoriatic arthritis had type 2 diabetes mellitus. Likewise, in Canadian patients with psoriatic arthritis, the prevalence of diabetes mellitus has been found to be 43% higher than in the general population.^30^ The notable exception is obesity, which is estimated to affect 36.5% of US adults,^25^ but in the current cohort was identified in 16.5% of patients.

The prevalence of obesity may be difficult to study in health claims databases. In our analysis and in the analysis reported by Merola *et al*,^31^ both using the MarketScan database, the results for obesity were lower than that reported elsewhere.^20–22^ In patients selected from the Toronto and Vancouver sites in the International Psoriasis and Arthritis Research Team database, the proportions of patients with obesity (≥30 kg/m^2^) in the psoriatic arthritis and general populations were 37% and 18%, respectively.^32^ Husted and colleagues^22^ reported an obesity rate of 30% in patients with psoriatic arthritis. Physicians may not include obesity as a diagnosis because it most likely is not the reason patients sought or paid for medical services at that visit.

The prevalence rate of acute MI in the psoriatic arthritis population in this study was 0.56%, and the incidence per 100 patient-years was 0.34; by contrast, the incidence of acute MI per 100 patient-years was greater among patients with psoriasis (0.49).^19^ In both psoriatic populations, the incidence of acute MI is greater than in the general US population (0.17%).^33^ Information reported in the literature is limited regarding the prevalence of MI in patients with psoriatic arthritis. In an analysis of the University of Toronto psoriatic arthritis clinical database, Gladman and colleagues^34^ reported 50 MIs in 648 registered patients (7.7%), including events before study entry. Feldman and coworkers^21^ reported the prevalence of acute MI in 0.57% (7/1230) of patients with psoriasis and comorbid psoriatic arthritis. In a prospective cohort study of patients with psoriatic arthritis, Eder *et al* 35 concluded that the level of disease activity and extent of systemic inflammation were independent factors of cardiovascular events, in addition to traditional cardiovascular risk factors. It has been reported in the literature that the use of tumour necrosis factor inhibitors may be associated with a reduced risk of adverse cardiovascular events, although the authors recognised that well-controlled randomised studies would be needed to evaluate the exact cardiovascular effects.^36^ It is interesting to note that, in the current analysis, use of biologics was greater in the psoriatic arthritis population than in the psoriasis population^19^ (table 2).

In this study, the prevalence of cerebrovascular disease in the psoriatic arthritis population was 7.3%, and the prevalence of any stroke was 0.47%. Merola *et al* 31 reported the prevalence of cerebrovascular disease ranged from 6.2% to 6.7% for patients with psoriatic arthritis and psoriasis, but stroke was not mentioned specifically. Similar to other comorbid conditions, the prevalence of stroke in patients with psoriatic arthritis and psoriasis was higher in our study than in the general US population (2.5%).^24^

In the analysis reported here, the prevalence of depression in the psoriatic arthritis population was 21%, which is greater than in the US general population (7.6%),^37^ but similar to that reported by Merola *et al* 31 in patients with psoriatic arthritis and moderate to severe psoriasis (23.2%) and in patients with psoriatic arthritis and minimal skin involvement (20.1%). The prevalence of major depression in our study ranged from 7% to 8%. McDonough and colleagues^38^ also observed a similar prevalence of depression among 306 patients with psoriatic arthritis attending psoriatic arthritis and dermatology clinics at Toronto Western Hospital.

For the current analysis, we chose to identify cases of malignancies based on a patient having only one claim with a malignancy-related diagnosis code. Prevalence of malignancies in the psoriatic arthritis population included 6.32%, 3.93% and 1.14% of patients with solid tumours (including melanoma), non-melanoma skin cancer (NMSC) and haematological malignancies, respectively. A prospective study by Rohekar *et al*,^39^ which reported the results from an additional 14-year analysis of the University of Toronto psoriatic arthritis clinical database, found that malignancy developed in 68 of 665 (10.2%) patients with psoriatic arthritis. Feldman and coworkers reported a prevalence of 2.2% (27/1230) for NMSC and 2.0% (25/1230) for other malignancies.^21^ In a study of 2970 patients with psoriatic arthritis included in the Consortium of Rheumatology Researchers of North America registry, the adjudicated incidence rates of NMSC, solid tumours and haematological tumours were 0.5% (15/2970; 0.21 per 100 patient-years), 0.67% (20/2970; 0.28 per 100 patient-years) and 0.17% (5/2970; 0.07 per 100 patient-years), respectively.^40^ The annual incidence of any type of malignancy in the general US population is estimated at 0.44%.^41^ Such findings suggest that the incidence of malignancy varies widely and depends on the approach to detecting and verifying such events. Differences in reported incidences may be due to differences in sample size, unknown registry inclusion/exclusion criteria, differences in provider type (eg, university-affiliated clinics vs all community practitioners) and coding inconsistencies. Notably, the lowest reported malignancy incidence rate in patients with psoriatic arthritis was described when events were adjudicated, perhaps indicating that accurate detection requires clinical verification and validation with a larger cohort. Given these discrepancies, further validation to identify true prevalence or incidence rates of malignancies in psoriatic arthritis is needed.

The prevalence of fibromyalgia (16.6%) in our study aligns with the study by Brikman and colleagues.^42^

Notable differences in comorbidity rates between patients with psoriatic arthritis in this study (n=94 302) and patients with psoriasis in an identically designed analysis of MarketScan data (n=469 097)^19^ have been observed for depression, type 2 diabetes mellitus, osteoporosis, serious infections, NMSC, suicide and suicidal ideation, fibromyalgia, ulcerative colitis, gout, non-alcoholic liver disease, acute MI, uveitis and major depression. Despite the greater published focus and research on depression associated with psoriasis, the higher rate of depression in patients with psoriatic arthritis compared with psoriasis based on the current analysis was also observed by McDonough and coworkers.^38^

Except for acute MI and NMSC, patients with psoriatic arthritis had higher rates of comorbidities than patients with psoriasis, although it is important to recognise that within the psoriasis population, some of the patients may also have had psoriatic arthritis.^19^ A potential factor associated with these differences may be the different treatment paradigms for the two conditions. Lim and Stern studied the relationship between skin cancer and ultraviolet B (UVB) therapy in a safety study of psoralen plus ultraviolet A therapy, concluding that for patients with high UVB exposure levels, UVB confers a modest increase in NMSC risk.^43^ Additional research is needed to evaluate whether psoriatic arthritis is an independent risk factor for acute MI and NMSC comorbidities.

### Limitations

While large retrospective databases such as the one used for the current analysis have broad cohorts of patients representing a real-world population, limitations must be considered; these limitations have been previously published.^44^ The selected populations may not be reflective of the age distribution of the psoriatic arthritis population because the MarketScan database contains only claims from a US commercially insured population and healthcare claims from individuals with Medicare supplemental insurance paid for by employers. In addition, the psoriasis population may include patients who also have claims for psoriatic arthritis. The claim information could be subject to misclassifications, as the primary purpose is reimbursement, leading to potentially inaccurate identification of patients’ conditions. There is no single correct definition of an outcome (ICD-9-CM codes used), and each definition has its own pros and cons, with some typically more sensitive or more specific than others. Some differences in the prevalence rates in our study compared with research conducted by others most likely are a result of differences in the study design, that is, selection of ICD-9-CM codes, study period and selection of diagnosis fields. The comparison of the comorbidity rates with the general US population based on references is a limitation of the analysis; it would have been best to have selected a control group from the MarketScan database. In the future, a database that coordinates medical records, such as the UK Clinical Practice Research Datalink data set, which contains high-quality longitudinal person-specific records that enable drug safety and outcomes research, may become available in the USA and would provide greater opportunities for outcome research in large population-based studies.

### Summary

This observational retrospective analysis of a large administrative health claims database assessed the real-world prevalence and incidence of comorbidities in a large cohort of patients diagnosed with psoriatic arthritis in the USA. The results are important, as information in the literature is limited regarding the prevalence and incidence of comorbidities in the US patient population with psoriatic arthritis, and real-world evidence will be helpful to healthcare providers who manage the care of these patients. These findings, which are consistent with other reports in the literature, demonstrate that a substantial proportion of patients with psoriatic arthritis have comorbid conditions that may confer additional health risks and may influence the choice of treatment.

## Conclusion

Health claims databases provide a unique opportunity and are a valuable resource for estimating the prevalence and incidence of comorbid diseases in large, diverse patient populations. Our study highlights the need to assess possible comorbid conditions in patients with psoriatic disease and to incorporate that information into treatment considerations and outcomes.

10.1136/rmdopen-2017-000588.supp2 Abstract translation This web only file has been produced by the BMJ Publishing Group from an electronic file supplied by the author(s) and has not been edited for content.
