# Supplementary material for: Real-world burden of comorbidities in US patients with psoriatic arthritis
Source: RMD Open. 2017 Dec 28;3(2):e000588. doi: 10.1136/rmdopen-2017-000588 (PMC5761305; doi:10.1136/rmdopen-2017-000588)
Supplement: Supplementary file 1 [file rmdopen-2017-000588supp001.docx]

**Supplementary Table 1** ICD-9-CM codes for outcomes

| Outcome | ICD-9-CM codes | Claims | Diagnosis |
| --- | --- | --- | --- |
| NMSC, excluding melanoma | 173.xx; excludes all patients with melanoma  ICD-9-CM codes 172.xx | All claims | Any diagnosis |
| Solid tumours | 140.xx, 141.xx, 142.xx, 143.xx, 144.xx, 145.xx, 146.xx, 147.xx, 148.xx, 149.xx, 150.xx, 151.xx, 152.xx, 153.xx, 154.xx, 155.xx, 156.xx, 157.xx, 158.xx, 159.xx, 160.xx, 161.xx, 162.xx, 163.xx, 164.xx, 165.xx, 170.xx, 171.xx, 172.xx, 174.xx, 175.xx, 176.xx, 179.xx, 180.xx, 181.xx, 182.xx, 183.xx, 184.xx, 185.xx, 186.xx, 187.xx, 188.xx, 189.xx, 190.xx, 191.xx, 192.xx, 193.xx, 194.xx, 195.xx, 196.xx, 197.xx, 198.xx, 199.xx, 209.0x, 209.1x, 209.2x and 209.3x | All claims | Any diagnosis |
| Haematological malignancies | 200.xx, 201.xx, 202.xx, 203.xx, 204.xx, 205.xx, 206.xx, 207.xx, 208.xx and 238.6 | All claims | Any diagnosis |
| Infections (1) | 254.1, 255.8, 323, 324, 357, 380.1, 382, 382.9, 383, 384, 384.1, 421, 422, 424.9, 447.2, 457.2, 460, 461, 462, 463, 464, 465, 466, 472, 473, 474, 475, 476, 480, 481, 482, 483, 484, 485, 486, 487.1, 487.8, 490, 4912, 510, 511.1, 513, 519.1, 519.2, 522.5, 522.7, 523.3, 528.3, 528.5, 529, 540, 541, 542, 550, 551, 566, 567, 5695, 572, 572.1, 574, 574.1, 574.3, 574.4, 575, 575.1, 576.1, 590, 595, 597, 5978, 599, 6031, 604, 607.1, 607.2, 614, 615, 615.9, 616, 680, 681, 682, 684, 685, 686, 711, 711.9, 728, 729.4, 730, 785.4, 788.7, 790.7, 790.8, 996.6, 998.5 and 999.3 | Inpatient claims | Any diagnosis |
| Major depression | 296.2 and 296.3 | All claims | Any diagnosis |
| Depression (2) | 296.2x, 296.3x, 300.4, 309.0, 309.1, 309.28, 311, 296.82 and 296.90 | All claims | All diagnoses |
| Suicide/suicidal ideation | E950, E951, E952, E953, E954, E955, E956, E957, E958 and V6284 | All claims | Any diagnosis |
| Acute MI (3) | 410.xx | Inpatient claims | Discharge diagnosis |
| Any stroke (4) | 430.xx, 431.xx, 433.01, 433.11, 433.21, 433.31, 433.81, 433.91, 434.01, 434.11, 431.91 and 436.xx | Inpatient claims | Any diagnosis |
| Ischemic stroke | 433.01, 433.11, 433.21, 433.31,433.81, 433.91, 434.01 , 434.11, 434.91 and 436.xx | Inpatient claims | Any diagnosis |
| Cerebrovascular diseases (5) | 430.xx, 431.xx, 432.xx, 433.xx, 434.xx, 435.xx, 436.xx, 437.xx and 438.xx | All claims | Any diagnosis |
| Congestive heart failure (5) | 428.xx | All claims | Any diagnosis |
| Type 2 diabetes mellitus (5) | 250.x0 and 250.x2 | All claims | Any diagnosis |
| Hyperlipidaemia (5) | 272.0x, 272.1x, 272.2x, 272.3x and 272.4x | All claims | Any diagnosis |
| Hypertension (5) | 401.x | All claims | Any diagnosis |
| Ischemic heart disease (5) | 410.xx, 411.xx, 412.xx, 413.xx and 414.xx | All claims | Any diagnosis |
| Peripheral vascular disease (5) | 440.xx, 441.xx, 443.xx, 447.1, 557.1, 557.9 and V43.4 | All claims | Any diagnosis |
| Atherosclerosis (5) | 440.xx | All claims | Any diagnosis |
| Osteoporosis | 733.0x, 733.00, 733.01, 733.02, 733.03, 733.09, 733.1x, 733.10, 733.11, 733.12, 733.13, 733.14, 733.15, 733.16 and 733.19 | All claims | Any diagnosis |
| Obesity | 278.0, 278.00, 278.01, 278.02 and 278.03 | All claims | Any diagnosis |
| Crohn’s disease | 555.9x | All claims | Any diagnosis |
| Uveitis | 364.3, 364.00, 364.02, 364.04 and 364.10 | All claims | Any diagnosis |
| Chronic renal insufficiency (6) | 582.xx, 583.xx, 585.xx, 586.xx and 587.xx | All claims | Any diagnosis |
| Cardiac dysrhythmias | 427.0x, 427.1x, 427.2x, 427.31, 427.32, 427.41, 427.42, 427.5x, 427.81, 427.89 and 427.9x | All claims | Any diagnosis |
| Fibromyalgia | 729.1 | All claims | Any diagnosis |
| Ulcerative colitis | 556.x | All claims | Any diagnosis |
| Gout | 274.xx | All claims | Any diagnosis |
| Other chronic  non-alcoholic liver disease | [571.8](http://www.icd9data.com/2015/Volume1/520-579/570-579/571/571.8.htm) | All claims | Any diagnosis |

ICD-9-CM, International Classification of Diseases, 9th Revision, Clinical Modification; MI, myocardial infarction; NMSC, non-melanoma skin cancers.

**References**

1. Nguyen-Khoa BA, Goehring EL Jr, Alexander KA, *et al*. Risk of significant infection in rheumatoid arthritis patients switching anti-tumor necrosis factor-alpha drugs. *Semin Arthritis Rheum* 2012;42:119-26.
2. Fiest KM, Jette N, Quan H, *et al*. Systematic review and assessment of validated case definitions for depression in administrative data. *BMC Psychiatry* 2014;14:289.
3. Metcalfe A, Neudam A, Forde S, *et al*. Case definitions for acute myocardial infarction in administrative databases and their impact on in-hospital mortality rates. *Health Serv Res* 2013;48:290-318.
4. Tirschwell DL, Longstreth WT Jr. Validating administrative data in stroke research. *Stroke* 2002;33:2465-70.
5. Kimball AB, Robinson D Jr, Wu Y, *et al*. Cardiovascular disease and risk factors among psoriasis patients in two US healthcare databases, 2001-2002. *Dermatology* 2008;217:27-37.
6. Winkelmayer WC, Schneeweiss S, Mogun H, *et al*. Identification of individuals with CKD from Medicare claims data: a validation study. *Am J Kidney Dis* 2005;46:225-32.

**Supplementary Table 2** Demographic characteristics of the US psoriatic arthritis population

| Characteristic | Psoriatic arthritis population (N = 94,302) | **Continuously** **enrolled population** **(N = 47,438)** |
| --- | --- | --- |
| Female, n (%) | 50,557 (53.6) | 25,891 (54.58) |
| Age, years |  |  |
| Mean | 50.9 | 51.1 |
| Median | 52 | 52 |
| Age grouping, n (%) |  |  |
| 18-40 years | 19,720 (20.91) | 9731 (20.51) |
| 41-65 years | 65,159 (69.10) | 32,672 (68.87) |
| >65 years | 9423 (9.99) | 5035 (10.61) |
| Diagnosis year, n (%) |  |  |
| 2008 | 6695 (7.10) | 5029 (10.60) |
| 2009 | 17,008 (18.04) | 8133 (17.14) |
| 2010 | 15,475 (16.41) | 7392 (15.58) |
| 2011 | 17,543 (18.60) | 8736 (18.42) |
| 2012 | 15,499 (16.44) | 7909 (16.67) |
| 2013 | 13,800 (14.63) | 6675 (14.07) |
| 2014 | 8282 (8.78) | 3559 (7.5) |
