# Supplementary material for: Real-world burden of comorbidities in US patients with psoriatic arthritis
Source: RMD Open. 2017 Dec 28;3(2):e000588. doi: 10.1136/rmdopen-2017-000588 (PMC5761305; doi:10.1136/rmdopen-2017-000588)
Supplement: Abstract translation [file rmdopen-2017-000588supp002.docx]

**ABSTRACT**

**Objectives** We assessed comorbidities associated with psoriatic arthritis in a broad cohort of US-insured adult patients using the Truven Health Analytics MarketScan^®^ database.

**Methods** Prevalence and incidence rates were assessed for 28 comorbid conditions among adult patients in the MarketScan database with a diagnosis of psoriatic arthritis and having two or more health claims for psoriatic arthritis between 1 July 2008 and 31 July 2015. Findings were compared with those of a similar, previously published analysis of patients with psoriasis.

**Results** Among a total of 186,552 patients with a diagnosis of psoriatic arthritis, 94,302 had two or more health claims for psoriatic arthritis during the study period and were included in the comorbidity analysis. The prevalence and incidence rates of the most common comorbidities were: hyperlipidaemia, 47.5% and 35.0%, respectively; hypertension, 47.3% and 31.3%; depression, 21.2% and 15.4%; type 2 diabetes mellitus, 20.2% and 13.5%; and fibromyalgia, 16.6% and 12.4%. Patients with psoriatic arthritis had notably higher incidence rates of uveitis, fibromyalgia, osteoporosis, Crohn’s disease and non-alcoholic liver disease than patients with psoriasis.

**Conclusion** This observational retrospective analysis using the MarketScan database provides real-world health claims data on the prevalence and incidence of comorbidities in a large US patient population with psoriatic arthritis. The observed high cardiometabolic comorbidity rates align with those reported in the literature and may help healthcare providers in the comprehensive management of patients with psoriatic arthritis.
